# Evidence-based medicine during the COVID-19 pandemic: A hematologist's perspective

**DOI:** 10.1016/j.htct.2025.103825

**Published:** 2025-04-05

**Authors:** Yung Gonzaga

**Affiliations:** Instituto Nacional de Câncer (INCA), Rio de Janeiro, Rio de Janeiro, Brazil

Dear Editor,

In December 2019, the first cases of a previously unknown pneumonia emerged in Wuhan, China. In January 2020, the causative agent was identified as a novel coronavirus, SARS-CoV-2.^1^ The virus quickly spread worldwide, and in March 2020, the World Health Organization declared it a pandemic. The rest is history.

I vividly remember the first patient admitted to our hematology-oncology ward with COVID-19 pneumonia. She was a young woman with acute myeloid leukemia in remission after her first cycle of induction chemotherapy. She presented with fever and nasal congestion, which quickly progressed to dry cough and shortness of breath. Upon admission, she was tachypneic, experiencing mild respiratory distress, and her oxygen saturation was 86% on room air, requiring supplemental oxygen. A chest computed tomography scan revealed bilateral ground-glass opacities involving >75% of her lung parenchyma - a radiological pattern that would soon become the hallmark of COVID-19 pneumonia,^2^ and a grim predictor of severe, potentially fatal, outcomes.

How do you apply the principles of evidence-based medicine (EBM) - the best available evidence, clinical expertise, and patient values - to guide decisions in managing a previously unknown disease? At the onset of the pandemic, no literature existed to inform clinical practice. By the end of 2020, however, nearly 95,000 articles on COVID-19 had flooded PubMed. Clinical experience had to be extrapolated from analogous conditions, while patient values were often reduced to a desperate plea: “Please, doctor, don't let me die.”

In June 2020, amidst an overwhelming influx of poor-quality studies, a large randomized clinical trial demonstrated that dexamethasone reduced 28-day mortality in hospitalized COVID-19 patients requiring oxygen or mechanical ventilation compared to standard care.^3^ Finally, there was evidence supporting a treatment that reduced mortality, utilizing an inexpensive, widely available, and well-known drug. Dexamethasone quickly became the global standard of care for these patients, likely saving thousands of lives at the pandemic's peak. One fundamental pillar of EBM - the best available evidence - was now accessible to guide clinical decisions. I could prescribe dexamethasone for my onco-hematologic patients with COVID-19 pneumonia to reduce their risk of death. Or could I?

Despite the trial's robustness and broad inclusion criteria, it did not include onco-hematologic patients. How applicable were the results to my patients, who were profoundly immunosuppressed due to their disease and treatments? Would initiating dexamethasone worsen their immunosuppression, exacerbating the viral infection or predisposing them to secondary infections and potentially fatal outcomes? While there was biological plausibility for both benefit and harm, high-quality evidence supported benefit. However, data on onco-hematologic patients - theoretically among the most vulnerable to increased immunosuppression - were lacking. With patients continuing to arrive, we could not wait for a trial specifically designed for hematologic malignancies. Decisions had to be made despite considerable uncertainty.

This scenario exemplifies an extreme application of the concept of external validity. It challenges the extent to which findings from a study's target population (general hospitalized COVID-19 patients) can be extrapolated to a distinct population (onco-hematologic patients with COVID-19). This process is neither statistical nor purely methodological; it is an intellectual exercise requiring specialized knowledge, clinical judgment, and decision-making in the face of uncertainty. Fully aware of the possibility of error, we decided to prescribe dexamethasone for our onco-hematologic patients hospitalized with COVID-19 pneumonia requiring oxygen support.

Time passed. We treated countless patients, celebrated successes, mourned losses, gathered data, and learned through practice. As vaccination campaigns took effect, hospital admissions declined, and cases generally became milder.^4^ With growing experience, we reflected on our decisions. Had prescribing dexamethasone been the right choice?

In 2024, a real-world observational study titled “Dexamethasone Treatment for COVID-19 is Associated with Increased Mortality in Patients with Hematologic Malignancies” was published.^5^ For those unfamiliar with critical appraisal of evidence, this finding may have been alarming, raising concerns about how many patients may have been harmed by our decision. However, for those well-versed in EBM principles, the study reinforced a crucial lesson: randomized controlled trials (RCTs) remain the gold standard for evaluating interventions. Random allocation ensures comparable groups, isolating the intervention's effect. Observational studies, in contrast, frequently reflect clinician-driven treatment decisions.^6^ In this case, sicker patients were more likely to receive dexamethasone, introducing confounding by indication - a scenario in which disease severity, rather than the intervention, determines the outcome.

To this day, we do not know whether dexamethasone helped, harmed, or had no effect on our patients. What we do know is that science - particularly through vaccines and a collective global effort - ultimately triumphed over the pandemic. During those challenging times, we made the best decisions we could with the information available, our clinical judgment, and an unwavering intent to help our patients.

## Conflicts of interest

The author declares no conflicts of interest.
